# Domain-based prediction of the human isoform interactome provides insights into the functional impact of alternative splicing

**DOI:** 10.1371/journal.pcbi.1005717

**Published:** 2017-08-28

**Authors:** Mohamed Ali Ghadie, Luke Lambourne, Marc Vidal, Yu Xia

**Affiliations:** 1 Department of Bioengineering, McGill University, Montreal, Québec, Canada; 2 Center for Cancer Systems Biology (CCSB), Dana-Farber Cancer Institute, Boston, Massachusetts, United States of America; 3 Department of Genetics, Harvard Medical School, Boston, Massachusetts, United States of America; University of Chicago, UNITED STATES

## Abstract

Alternative splicing is known to remodel protein-protein interaction networks (“interactomes”), yet large-scale determination of isoform-specific interactions remains challenging. We present a domain-based method to predict the isoform interactome from the reference interactome. First, we construct the domain-resolved reference interactome by mapping known domain-domain interactions onto experimentally-determined interactions between reference proteins. Then, we construct the isoform interactome by predicting that an isoform loses an interaction if it loses the domain mediating the interaction. Our prediction framework is of high-quality when assessed by experimental data. The predicted human isoform interactome reveals extensive network remodeling by alternative splicing. Protein pairs interacting with different isoforms of the same gene tend to be more divergent in biological function, tissue expression, and disease phenotype than protein pairs interacting with the same isoforms. Our prediction method complements experimental efforts, and demonstrates that integrating structural domain information with interactomes provides insights into the functional impact of alternative splicing.

## Author summary

Protein-protein interaction networks have been extensively used in systems biology to study the role of proteins in cell function and disease. However, current network biology studies typically assume that one gene encodes one protein isoform, ignoring the effect of alternative splicing. Alternative splicing allows a gene to produce multiple protein isoforms, by alternatively selecting distinct regions in the gene to be translated to protein products. Here, we present a computational method to predict and analyze the large-scale effect of alternative splicing on protein-protein interaction networks. Starting with a reference protein-protein interaction network determined by experiments, our method annotates protein-protein interactions with domain-domain interactions, and predicts that a protein isoform loses an interaction if it loses the domain mediating the interaction as a result of alternative splicing. Our predictions reveal the central role of alternative splicing in extensively remodeling the human protein-protein interaction network, and in increasing the functional complexity of the human cell. Our prediction method complements ongoing experimental efforts by predicting isoform-specific interactions for genes not tested yet by experiments and providing insights into the functional impact of alternative splicing.

## Introduction

Protein-protein interaction (PPI) networks (also known as interactome networks) have been extensively studied in systems biology to understand genotype-phenotype relationships and several have been constructed for different model organisms such as human, yeast and bacteria [1–10]. The increase in the number of interactions reported by independent studies has led to the construction of large databases of experimentally determined PPIs, such as IntAct [11] and BioGRID [12]. In the case of human interactome mapping, some studies have used systematic yeast two-hybrid (Y2H) screening to generate large-scale, high-quality maps of the human binary interactome network [13], whereas other studies have used mass spectrometry to generate catalogues of protein complexes in human cells [14, 15]. The utility of these PPI networks can be further enhanced by annotating nodes and edges with structural domain information [16–21]. Despite their success, current network biology studies typically make the assumption that one gene encodes one protein isoform, and ignore the effect of alterative splicing (AS).

It is estimated that more than 100,000 AS events occur in pre-mRNA transcripts of human multi-exon genes [22, 23] and that over two-thirds of human genes contain one or more alternatively spliced exons [22, 24, 25]. During evolution, the expansion of the proteome by AS correlates positively with the increase in species complexity [26, 27]. In human, splicing events occur frequently in a tissue specific manner [22, 28] and in regions located on the surfaces of proteins, which are candidates for mediating molecular interactions [29, 30]. Moreover, AS occurs more often in transcripts which encode proteins that are involved in a high number of interactions and, through alternative inclusion or exclusion of exons, creates or eliminates protein-protein interactions [28, 31]. Given the known impact of AS on protein function [32], and its strong association to disease [33], efforts to systematically relate this functional impact to its role in remodeling the human interactome recently culminated in the large-scale mapping of a human isoform interactome [34]. Subsequent analysis of this experimentally mapped isoform interactome showed that different isoforms of the same gene having different interaction profiles with other proteins tend to behave as products of different genes in terms of function, disease phenotype and tissue expression, proving that AS contributes to the functional complexity of different human cell types by creating or eliminating isoform interactions. However, due to the challenging nature of these experiments, the current human isoform interactome is far from complete where protein isoforms encoded by less than 5% of the human genome were successfully tested for PPIs. Hence, there is a great need to complement experimental efforts with the development of computational methods for accurate prediction of isoform interactions. Such computational predictions also enable us to assess the general applicability of insights gained from the size-limited experimental datasets on the human isoform interactome.

**Fig 1:**
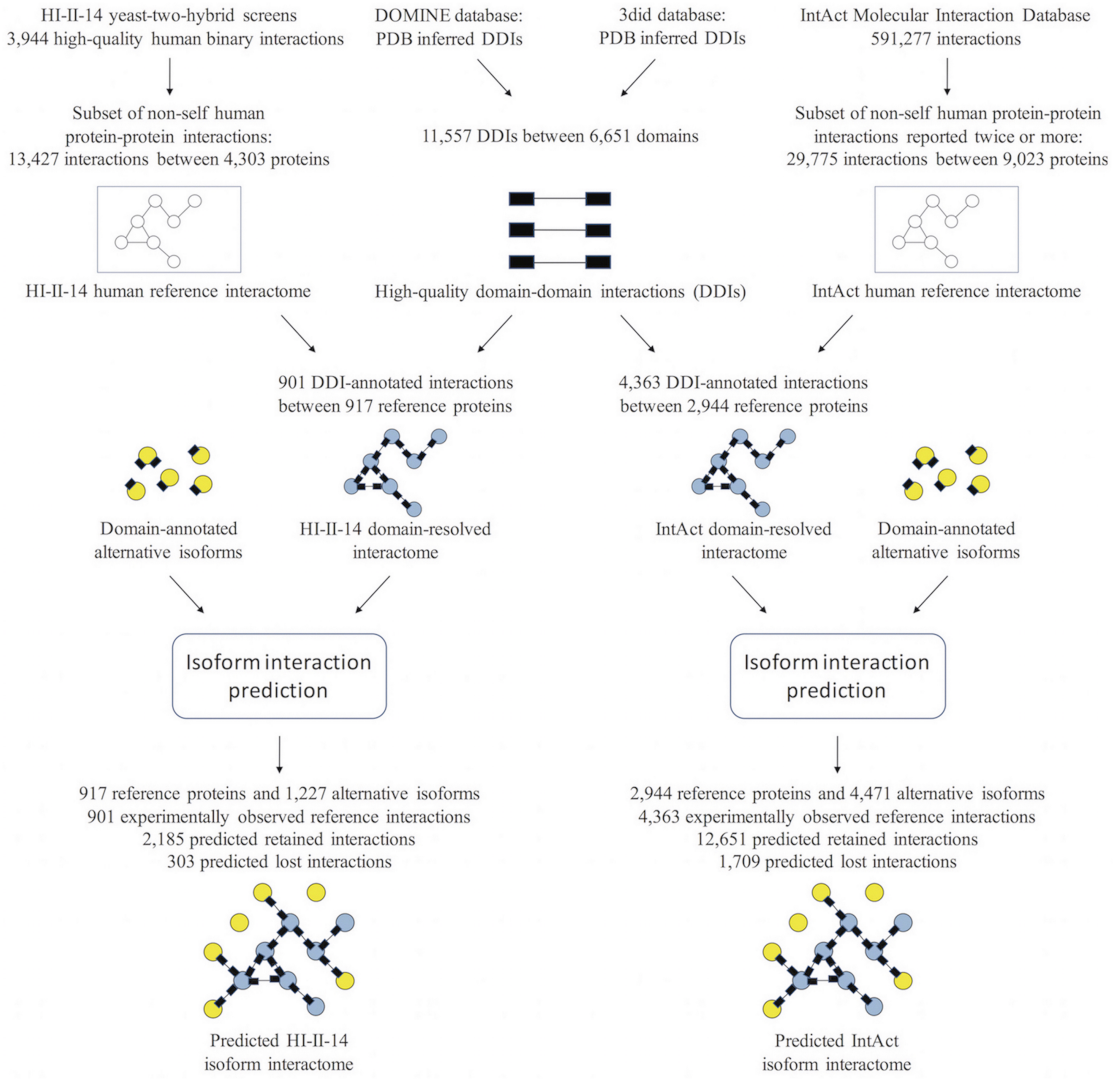
Computational pipeline for the prediction of the human isoform interactome. Domain-domain interactions are mapped onto two human reference interactomes, HI-II-14 [13] and IntAct [11]. The resulting domain-resolved reference interactomes are then used to predict interactions for the alternative isoforms of proteins in these interactomes, resulting in two predicted isoform interactomes.

In this paper we present a computational method, named DIIP: Domain-based Isoform Interactome Prediction, that predicts the isoform interactome from an experimentally determined reference interactome, with application to human (Fig 1). Starting with experimentally determined interactions between reference proteins, we map known structural domains onto proteins and known domain-domain interactions (DDIs) onto PPIs, and construct a domain-resolved reference interactome where PPIs are annotated with DDIs. Next, we construct an isoform interactome by expanding this domain-resolved reference interactome to include interactions predicted for alternative isoforms of reference proteins. Specifically, for each interaction involving a reference protein, an alternative isoform is predicted to maintain the interaction if the DDI mediating the interaction is retained, and predicted to lose the interaction if the DDI mediating the interaction is lost. We apply our computational method to predict isoform interactomes from two human reference interactomes, the high-quality HI-II-14 reference binary interactome [13] and the larger interactome from IntAct [11]. We find extensive network remodeling by alternative splicing: ~22% of genes with two or more isoforms in the predicted isoform interactome have at least one isoform losing an interaction, and ~18% of isoform pairs encoded by the same gene in the isoform interactome have different interaction profiles. In addition, we find that compared to protein pairs interacting with the same subset of isoforms of the same gene, protein pairs interacting with different subsets of isoforms of the same gene tend to be more divergent in terms of function, disease phenotype and tissue expression. Our predicted isoform interactome explores a different part of the isoform space than the experimentally mapped isoform interactome of Yang et al. (2016) [34]. Despite this minimal overlap, our results are consistent with the results of Yang et al. (2016), indicating that these results are broadly applicable to the human isoform interactome. Finally, using the experimentally mapped interactome of Yang et al. (2016) as a benchmark dataset, we show that our computational framework for predicting isoform interactions is of high-quality, performing better than random expectation. All together, our results show that our computational method complements experimental efforts, and that integrating structural domain information with PPI networks provides insights into the functional impact of AS on different human cell types through remodeling of the human interactome network.

## Results

### Predicted human isoform interactome reveals extensive network remodeling by AS

We used the 11,557 DDIs combined from the 3did database of three-dimensional interacting domains [35] and the DOMINE Database of Protein Domain Interactions [36] which were inferred from Protein Data Bank (PDB) entries to construct two domain-resolved reference interactomes for human. The first domain-resolved reference interactome was constructed by annotating with DDIs the high-quality HI-II-14 human reference binary interactome [13] which consists of 13,427 interactions between 4,303 proteins. The resulting domain-resolved interactome consists of 917 proteins and 901 annotated interactions (S1 Table). The second domain-resolved reference interactome was constructed by annotating with DDIs the larger human interactome from IntAct [11] (retrieved May 2016) which consists of 9,023 proteins and 29,775 interactions reported by two or more experiments. The resulting domain-resolved reference interactome consists of 2,944 proteins and 4,363 annotated interactions (S2 Table).

From each domain-resolved reference interactome, we predicted an isoform interactome that includes experimentally determined interactions between reference proteins, as well as predicted interactions for the alternative isoforms of these proteins, where the isoform data are obtained from UniProt [37]. We predicted isoform interactions using the following rule: Given an experimentally determined interaction between two reference proteins annotated with one or more DDIs, we predict that an alternative isoform of one protein loses its interaction with the other protein if the isoform interaction loses all of the above-mentioned DDI annotations, otherwise the interaction is predicted to be retained. The predicted HI-II-14 isoform interactome consists of the 901 experimentally determined reference interactions, 2,185 predicted retained interactions and 303 predicted lost interactions involving the 917 reference proteins and their 1,227 alternative isoforms (S3 Table), whereas the predicted IntAct isoform interactome consists of the 4,363 experimentally determined reference interactions, 12,651 predicted retained interactions and 1,709 predicted lost interactions involving the 2,944 reference proteins and their 4,471 alternative isoforms (S4 Table). The lost interactions are spread among a large number of genes: 22.4% (130 of 580) of genes with two or more isoforms in the predicted HI-II-14 isoform interactome have at least one isoform losing one or more interactions, whereas 21% (402 of 1,911) of genes with two or more isoforms in the predicted IntAct isoform interactome have at least one isoform losing one or more interactions. Widespread remodeling of the human interactome by AS can also be seen at the level of isoform pairs. 18.8% of isoform pairs encoded by the same gene in the predicted HI-II-14 isoform interactome have different interaction profiles, whereas 16.5% of isoform pairs encoded by the same gene in the predicted IntAct isoform interactome have different interaction profiles. Altogether, these observations highlight the extensive role of AS in preserving or eliminating isoform interactions, hence creating different interaction profiles for different isoforms of the same gene.

**Fig 2:**
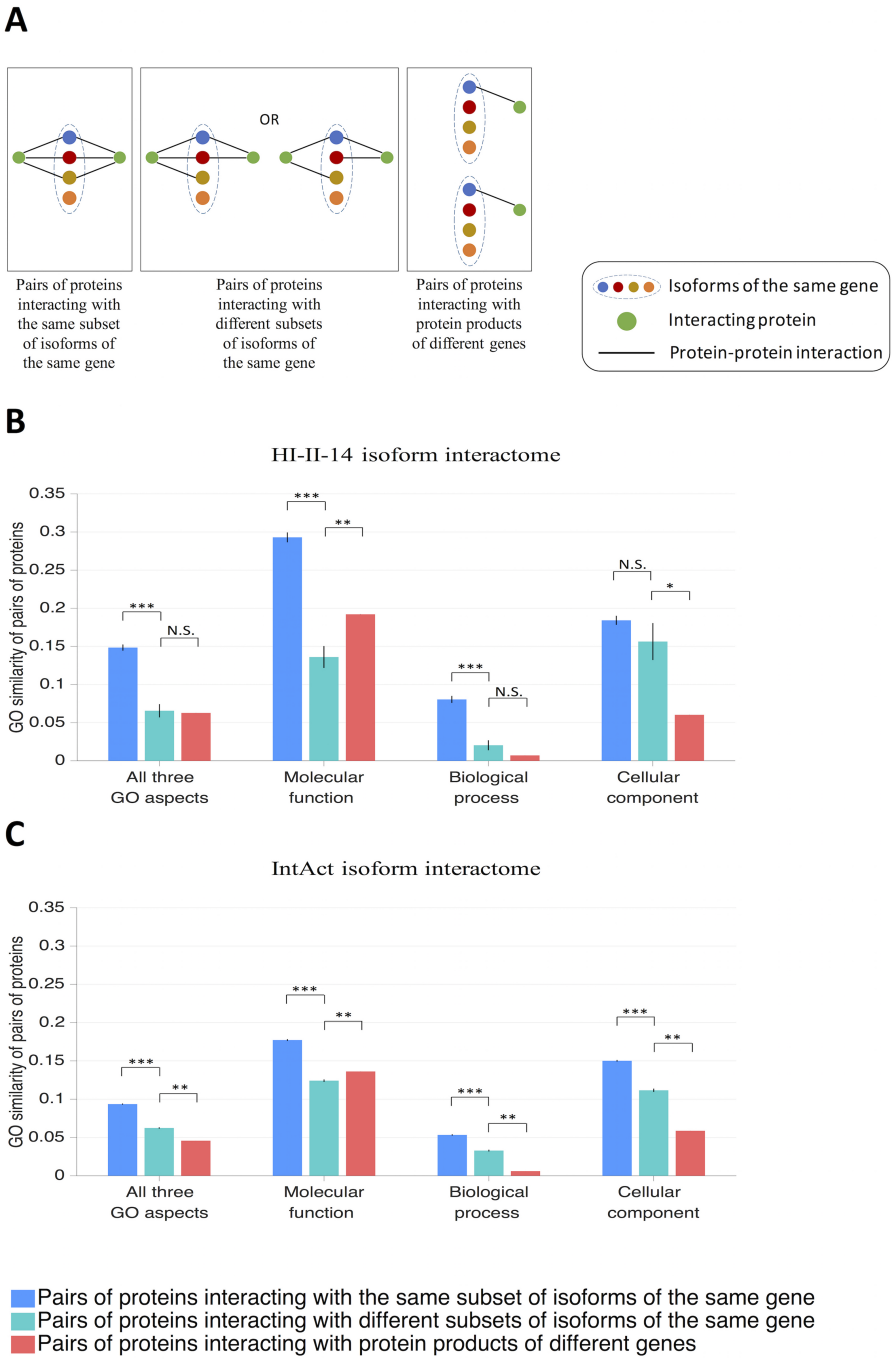
Gene Ontology (GO) similarity for protein pairs with different types of isoform interaction profiles. (A) Schematic diagram illustrating different types of isoform interaction profiles for a pair of proteins. (B) GO similarity for pairs of proteins interacting with the same subset of isoforms of the same gene, with different subsets of isoforms of the same gene, and with protein products of different genes in the predicted HI-II-14 isoform interactome. (C) GO similarity for pairs of proteins interacting with the same subset of isoforms of the same gene, with different subsets of isoforms of the same gene, and with protein products of different genes in the predicted IntAct isoform interactome. GO similarity was calculated using the Jaccard similarity index. Pairs in which both proteins have no GO annotations were excluded. Error bars represent standard errors of the mean. Statistical significance was calculated using a two-sided bootstrap test (*: p < 10^−2^, **: p < 10^−3^, ***: p < 10^−5^).

Next, we focused on protein pairs interacting with the same target protein in the reference interactome, and classified these protein pairs into those interacting with the same subset of isoforms of the same target gene, and those interacting with different subsets of isoforms of the same target gene (Fig 2A). The vast majority of protein pairs considered here belong to only one category; protein pairs belonging to more than one category for different target genes are extremely rare, and are excluded from further analysis. In the predicted HI-II-14 isoform interactome, we identified 1,437 protein pairs interacting with the same subset of isoforms of the same gene, and 63 protein pairs interacting with different subsets of isoforms of the same gene. In the predicted IntAct isoform interactome, we identified 20,685 protein pairs interacting with the same subset of isoforms of the same gene, and 2,669 protein pairs interacting with different subsets of isoforms of the same gene. Therefore, AS is capable of creating a wide variety of isoform interaction profiles for proteins interacting with the same target protein.

### Divergence in biological function of protein pairs interacting with different subsets of isoforms of the same gene

We retrieved Gene Ontology (GO) associations from the UniProt-GOA database [38] and constructed a GO association profile for each protein in the reference interactome. We then used the Jaccard similarity index to calculate GO similarity for each protein pair in which at least one protein is GO annotated. Finally, we systematically calculated and compared GO similarity for different types of protein pairs: those interacting with the same subset of isoforms of the same gene, those interacting with different subsets of isoforms of the same gene (as defined in the previous section), as well as those interacting with protein products of different genes only.

In the HI-II-14 isoform interactome, compared to protein pairs interacting with the same subset of isoforms of the same gene, protein pairs interacting with different subsets of isoforms of the same gene are less similar in molecular function, biological process, as well as all three GO categories combined (p < 10^−5^ each), but the similarity is not significantly different in cellular component (p = 0.25) (two-sided bootstrap test with 100,000 resamplings; Fig 2B). On the other hand, compared to protein pairs interacting with protein products of different genes, protein pairs interacting with different subsets of isoforms of the same gene are more similar in cellular component (p = 2 x 10^−3^), but less similar in molecular function (p < 10^−3^). However, the difference is not statistically significant in biological process (p = 0.054) and all three GO categories combined (p = 0.71) (two-sided bootstrap test with 1,000 resamplings; Fig 2B). In the IntAct isoform interactome, compared to protein pairs interacting with the same subset of isoforms of the same gene, protein pairs interacting with different subsets of isoforms of the same gene are less similar in molecular function, biological process, cellular component, as well as all three GO categories combined (p < 10^−5^ each, two-sided bootstrap test with 100,000 resamplings; Fig 2C). On the other hand, compared to protein pairs interacting with protein products of different genes, protein pairs interacting with different subsets of isoforms of the same gene are more similar in biological process, cellular component, as well as all three GO categories combined, but less similar in molecular function (p < 10^−3^ each, two-sided bootstrap test with 1,000 resamplings; Fig 2C).

Altogether, our results show that protein pairs interacting with different subsets of isoforms of the same gene tend to be more divergent in biological function than protein pairs interacting with the same subset of isoforms of the same gene. In addition, they tend to be less divergent in biological function than protein pairs interacting with protein products of different genes, albeit to a lesser degree. Notably, for the GO molecular function aspect, protein pairs interacting with different subsets of isoforms of the same gene can be as divergent as protein pairs interacting with protein products of different genes. Our results therefore demonstrate that AS increases the functional complexity of human cells by remodeling the human interactome network.

### Divergence in disease phenotype of protein pairs interacting with different subsets of isoforms of the same gene

Disruptions in AS events in human are associated with a wide range of known diseases [33]. To investigate the extent to which protein pairs interacting with different subsets of isoforms of the same gene are associated with different disease phenotypes, we retrieved gene-disease associations from the DisGeNET database [39, 40] and constructed a disease annotation profile for each human reference protein. Because the fraction of human proteins with disease annotations is small, we further constructed a disease subnetwork profile for each human reference protein where a protein belongs to a specific disease subnetwork if that protein or any of its interaction partners in the unbiased, high-quality HI-II-14 reference binary interactome is annotated with the disease. We then used the Jaccard similarity index to calculate the fraction of disease subnetworks shared by pairs of reference proteins each annotated with at least one disease subnetwork, where two proteins share a specific disease subnetwork if each of the two proteins or any of its interaction partners in the HI-II-14 reference binary interactome is annotated with that disease. Finally, we systematically calculated and compared disease subnetwork sharing for different types of protein pairs: those interacting with the same subset of isoforms of the same gene, those interacting with different subsets of isoforms of the same gene (as defined previously), and those interacting with protein products of different genes only.

**Fig 3:**
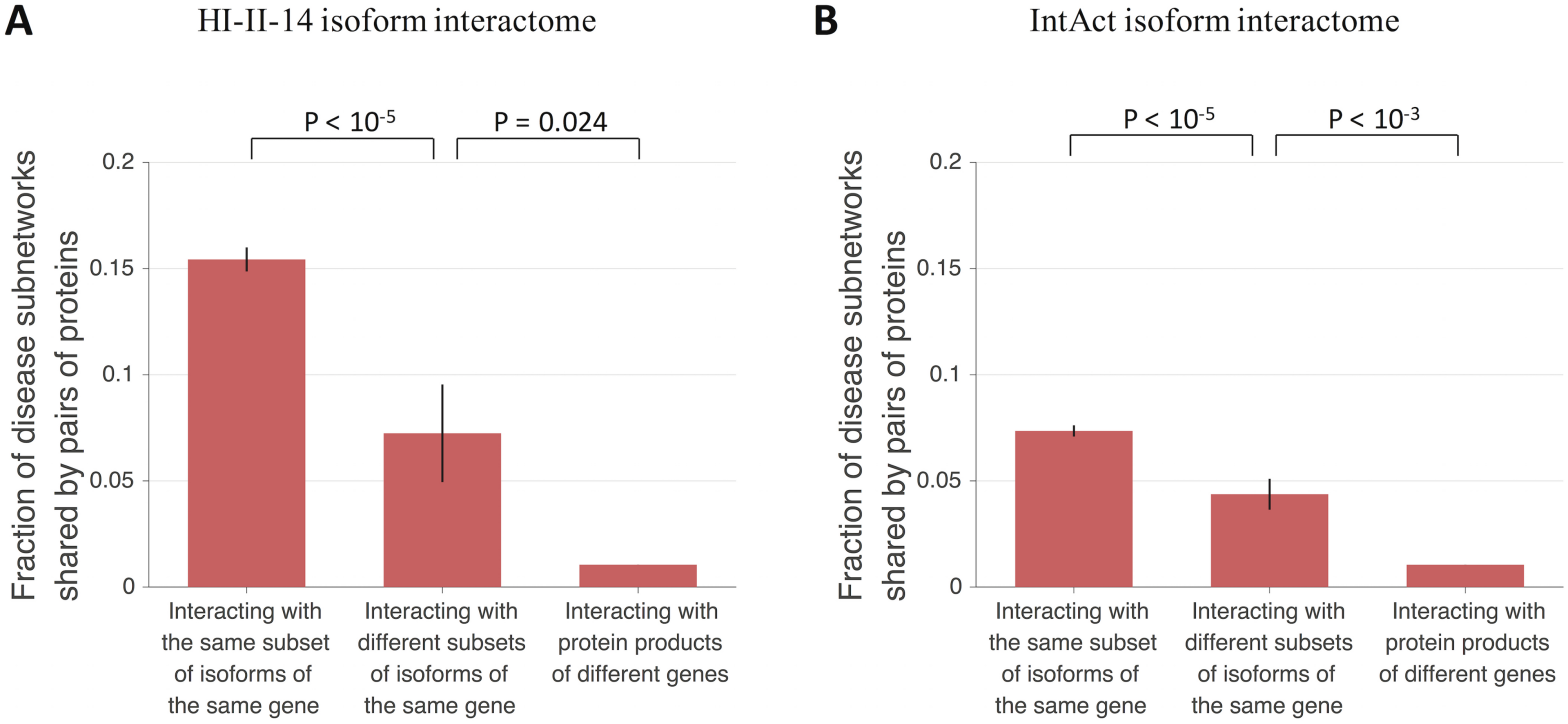
Fraction of shared disease subnetworks for protein pairs with different types of isoform interaction profiles. (A) Fraction of shared disease subnetworks for pairs of proteins interacting with the same subset of isoforms of the same gene, with different subsets of isoforms of the same gene, and with protein products of different genes in the predicted HI-II-14 isoform interactome. (B) Fraction of shared disease subnetworks for pairs of proteins interacting with the same subset of isoforms of the same gene, with different subsets of isoforms of the same gene, and with protein products of different genes in the predicted IntAct isoform interactome. Fraction of shared disease subnetworks was calculated using the Jaccard similarity index. Only pairs in which both proteins are associated with at least one disease subnetwork were considered. A protein belongs to a disease subnetwork if that protein or any of its first-degree neighbors in the HI-II-14 reference binary interactome are associated with the disease. Error bars represent standard errors of the mean. Statistical significance was calculated using a two-sided bootstrap test.

In the HI-II-14 isoform interactome, protein pairs interacting with different subsets of isoforms of the same gene (n = 47) tend to share a smaller fraction of disease subnetworks than protein pairs interacting with the same subset of isoforms of the same gene (n = 1,241) (p < 10^−5^, two-sided bootstrap test with 100,000 resamplings; Fig 3A). In addition, protein pairs interacting with different subsets of isoforms of the same gene tend to share a larger fraction of disease subnetworks than protein pairs interacting with protein products of different genes (p = 0.024, two-sided bootstrap test with 1,000 resamplings; Fig 3A). In the IntAct isoform interactome, protein pairs interacting with different subsets of isoforms of the same gene (n = 196) also tend to share a smaller fraction of disease subnetworks than protein pairs interacting with the same subset of isoforms of the same gene (n = 3,226) (p < 10^−5^, two-sided bootstrap test with 100,000 resamplings; Fig 3B), as well as a larger fraction of disease subnetworks than protein pairs interacting with protein products of different genes (p < 10^−3^, two-sided bootstrap test with 1,000 resamplings; Fig 3B).

All together, our results show that compared to protein pairs with identical isoform interaction profiles, protein pairs with different isoform interaction profiles tend to be more divergent in disease phenotype, consistent with the experimental results of Yang et al. (2016) [34]. Our results therefore demonstrate that by remodeling the human interactome network, AS creates divergence in disease phenotype among protein pairs interacting with different isoforms of the same gene.

### Divergence in tissue expression of protein pairs interacting with different subsets of isoforms of the same gene

**Fig 4:**
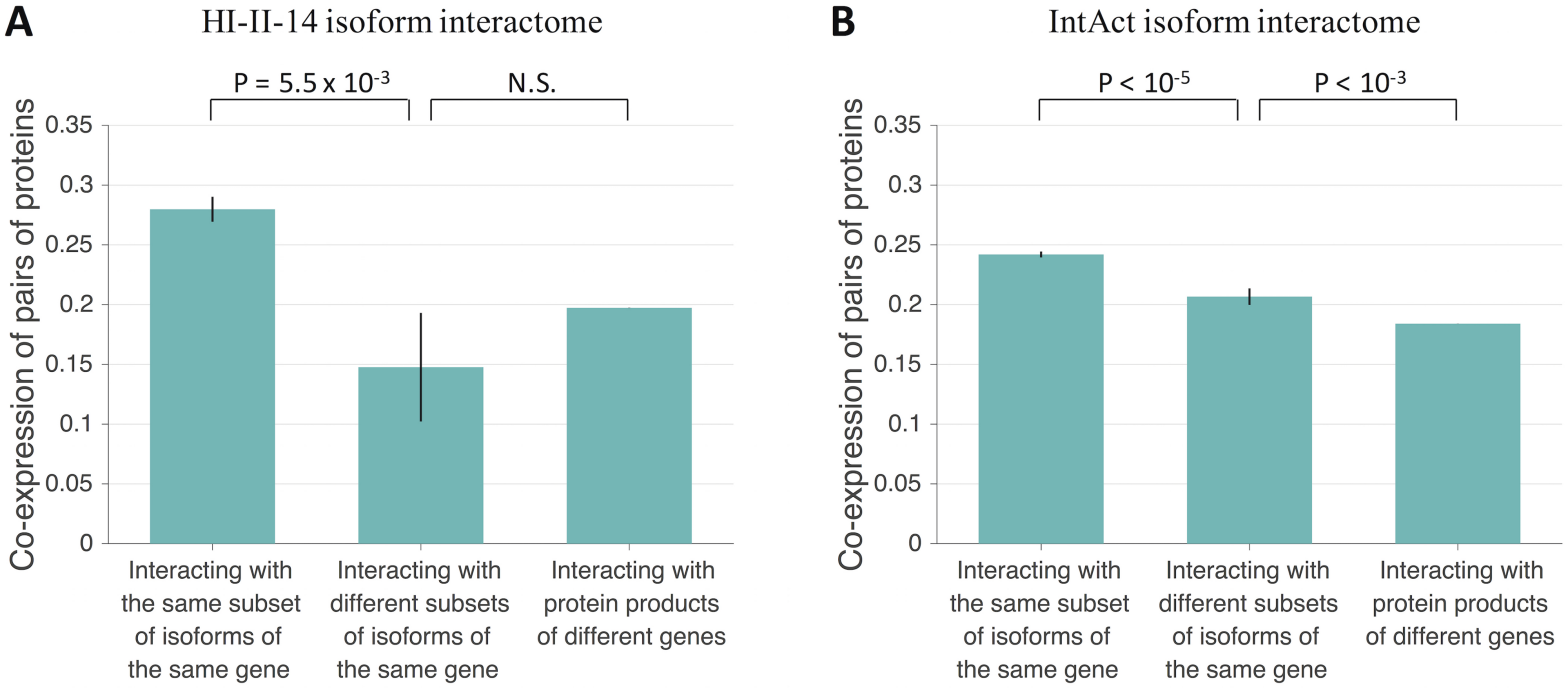
Tissue co-expression for protein pairs with different types of isoform interaction profiles. (A) Tissue co-expression for pairs of proteins interacting with the same subset of isoforms of the same gene, with different subsets of isoforms of the same gene, and with protein products of different genes in the predicted HI-II-14 isoform interactome. (B) Tissue co-expression for pairs of proteins interacting with the same subset of isoforms of the same gene, with different subsets of isoforms of the same gene, and with protein products of different genes in the predicted IntAct isoform interactome. Tissue co-expression was calculated using Pearson’s correlation coefficient. Only pairs in which both proteins with expression levels simultaneously quantified in at least 8 tissues were considered. Error bars represent standard errors of the mean. Statistical significance was calculated using a two-sided bootstrap test.

Since tissue expression strongly correlates with biological function and disease phenotype [41, 42], we investigated the extent to which protein pairs interacting with different subsets of isoforms of the same gene diverge in tissue expression, in addition to divergence in biological function and disease phenotype. We used the Illumina Body Map 2.0 RNA-Seq dataset [43] to quantify gene expression in 16 different body tissues, and calculated tissue co-expression using Pearson’s correlation coefficient for pairs of reference proteins with both expression levels simultaneously quantified in at least 8 tissues. In the HI-II-14 isoform interactome, protein pairs interacting with different subsets of isoforms of the same gene (n = 62) tend to be less co-expressed than protein pairs interacting with the same subset of isoforms of the same gene (n = 1,182) (p = 5.5 x 10^−3^, two-sided bootstrap test with 100,000 resamplings; Fig 4A). In the IntAct isoform interactome, protein pairs interacting with different subsets of isoforms of the same gene (n = 2,609) also tend to be less co-expressed than protein pairs interacting with the same subset of isoforms of the same gene (n = 19,678) (p < 10^−5^, two-sided bootstrap test with 100,000 resamplings; Fig 4B), and more co-expressed than protein pairs interacting with protein products of different genes (p < 10^−3^, two-sided bootstrap test with 1,000 resamplings; Fig 4B). Our results show that protein pairs interacting with different subsets of isoforms of the same gene tend to be more divergent in tissue expression than protein pairs interacting with the same subset of isoforms of the same gene, consistent with the experimental results of Yang et al. (2016) [34].

### Case studies of protein pairs predicted to interact with different subsets of isoforms of the same gene

**Fig 5:**
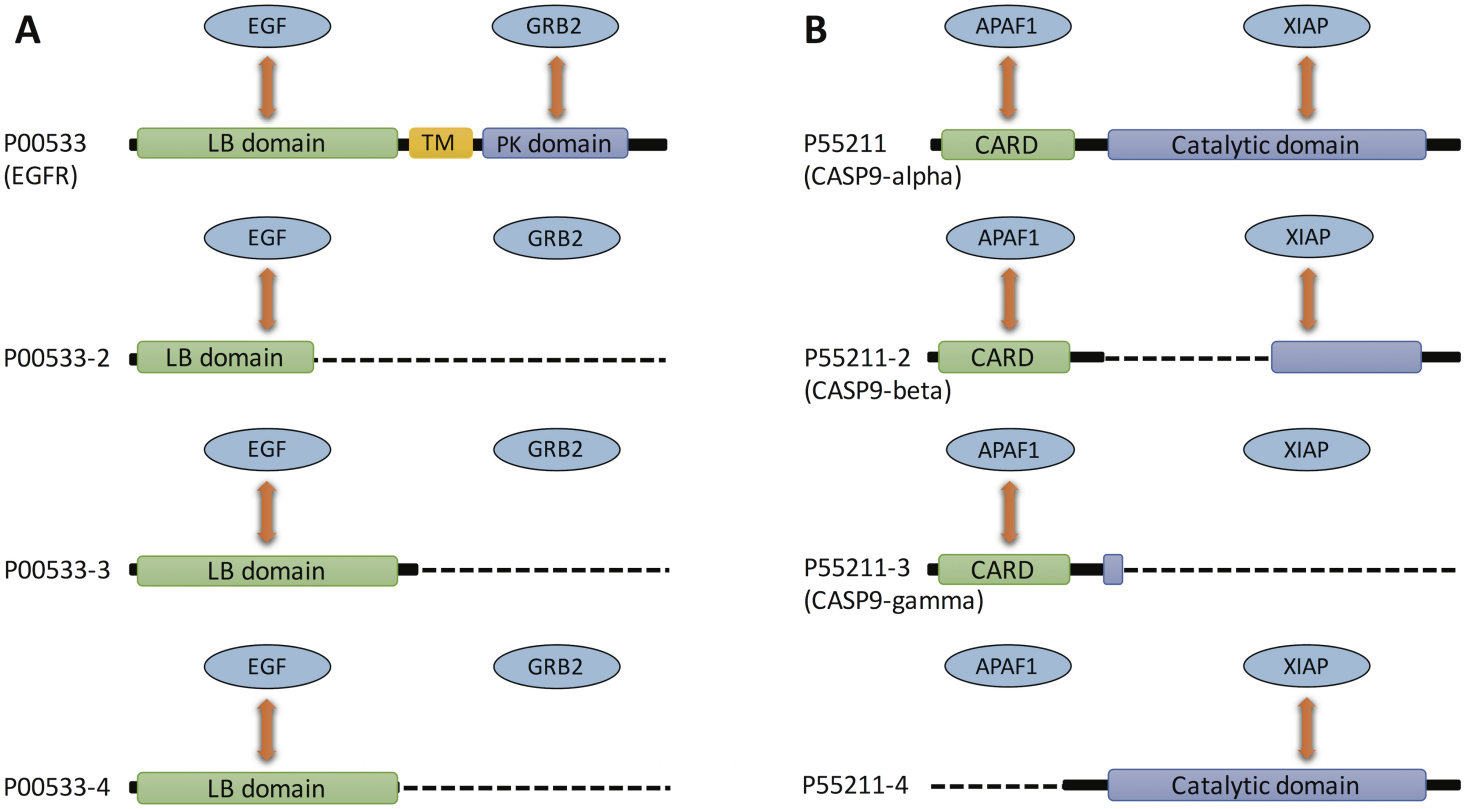
Case studies of protein pairs interacting with different subsets of isoforms of the same gene. (A) Two proteins (EGF and GRB2) interacting with different subsets of isoforms of EGFR. EGF interacts with all four isoforms of EGFR on their ligand-binding domain, whereas GRB2 interacts only with the first isoform (P00533) and does not interact with any of the other three isoforms (P00533-2, P00533-3 and P00533-4) that lack the protein kinase domain. (B) Two proteins (XIAP and APAF1) interacting with different subsets of isoforms of CASP9. APAF1 interacts with three isoforms of CASP9 (P55211, P55211-2 and P55211-3) on their CARD domain and loses interaction with the fourth isoform P55211-4. XIAP interacts with three isoforms of CASP9 (P55211, P55211-2 and P55211-4) on their catalytic domain and has no experimentally confirmed interaction with the third isoform P55211-3.

Here, we present case studies of two types of protein pairs predicted by our method to interact with different subsets of isoforms of the same gene. We first looked at the epidermal growth factor EGF and the growth factor receptor-bound protein GRB2, which were predicted by our method to interact with different subsets of isoforms of the epidermal growth factor receptor EGFR (UniProt ID: P00533) (Fig 5A). Our method predicted that EGF interacts with EGFR (P00533) and its three shorter alternative isoforms (P00533-2, P00533-3, P00533-4), whereas GRB2 was predicted to interact with EGFR (P00533) and lose interaction with all its alternative isoforms. All of these predictions are consistent with experiments. By interacting with EGFR, EGF and GRB2 carry out different functions in protein signaling. EGF activates P00533 by binding to its extracellular ligand binding (LB) domain, hence inducing autophosphorylation of its protein kinase (PK) domain [44]. GRB2 binds to the tyrosine-phosphorylated PK domain of P00533 through its SH3 domain, hence connecting growth factor stimulation to other intracellular signalling pathways [45]. P00533 consists of three main parts: the extracellular domain (exons 1–16) containing the LB domain, the transmembrane (TM) domain (exons 16–18), and the intracellular domain (exons 18–24) containing the PK domain. The second isoform (P00533-2) contains a large part of the extracellular domain which retains LB function [46], whereas the third and fourth isoforms (P00533-3 and P00533-4) contain the whole extracellular domain [47]. All three alternative isoforms of P00533, however, lack the TM domain and the intracellular domain. Therefore, EGF interacts with all four isoforms of EGFR on their LB domains, whereas GRB2 interacts with P00533 on its PK domain and loses interaction with the other three truncated isoforms. These three truncated isoforms have different tissue expression patterns [48], are expressed in different cancers [49–51], and may play a role in supressing cell growth by inhibiting EGFR [50, 51].

We also looked at the protein pair XIAP and APAF1 that were predicted by our method to interact with different subsets of isoforms of CASP9 (UniProt ID: P55211) (Fig 5B). Our method predicted that APAF1 interacts with CASP9 (P55211) and its alternative isoforms P55211-2 and P55211-3, and loses interaction with P55211-4. All of these predictions are consistent with experiments. Our method also predicted that XIAP interacts with CASP9 (P55211) and its alternative isoforms P55211-2, P55211-3 and P55211-4. All of these predictions are supported by experiments with the exception of XIAP’s zinc finger-mediated interaction with P55211-3, which retains the CARD domain known to interact with zinc fingers [52]. XIAP and APAF1 are known to carry out antagonistic functions in activating and inhibiting apoptosis. This is also true for their CASP9 isoform partners. The CASP9 gene encodes four isoforms: P55211 (CASP9-alpha) which has the longest sequence (416 residues), P55211-2 (CASP9-beta) which lacks a central large sequence segment (residues 140–289), P55211-3 (CASP9-gamma) which lacks the catalytic domain (residues 139–416) and only has the caspase recruitment domain (CARD) (residues 1–92), and P55211-4 that lacks the CARD domain but has the catalytic domain. XIAP inhibits apoptosis by binding to the catalytic domain of P55211 through its BIR3 domain thus inhibiting its catalytic activity [53]. On the other hand, APAF1 activates apoptosis by forming an apoptosome complex with P55211 through CARD-CARD interaction [54]. The isoforms of CASP9 also play different roles in apoptosis due to their different interaction profiles. P55211-3 which lacks the catalytic domain containing the active site for catalysis interacts with APAF1 through its CARD domain, interfering with the formation of the apoptosome and therefore functioning as an endogenous inhibitor of apoptosis [55]. Similarly, P55211-2 functions as an endogenous inhibitor of apoptosis by interacting with APAF1 through its CARD domain while at the same time losing a large part of its catalytic domain, even though it retains some residual interaction with XIAP [56]. On the other hand, P55211-4, which lacks the CARD domain, interacts with XIAP but does not interact with APAF1, suggesting that it may not inhibit apoptosis. Moreover, the interaction of P55211-4 with the apoptosis inhibitor XIAP suggests that it may promote apoptosis, contrary to P55211-2 and P55211-3.

Overall, these two case studies highlight specific mechanisms for how AS-mediated remodeling of interactions of the isoforms of the same gene leads to divergence in their biological function and disease phenotype, and illustrate that our computational method is capable of identifying biologically relevant isoform-specific interactions.

### Quality assessment of the isoform-interaction prediction method

Here, we empirically assessed the quality of our isoform-interaction prediction method by validating it against the experimental dataset of Yang et al. (2016) [34], which is the only genome-wide isoform interactome dataset available so far. However, the experimental dataset of Yang et al. (2016) is small in size for our purpose of validation: there are only 310 reference interactions between reference proteins, among which we were able to annotate only 34 reference interactions with known DDIs. We then predicted isoform-specific interactions from these 34 DDI-annotated reference interactions using our method, and compared our predictions with experiments. In terms of predicting interaction loss events, we obtained a true positive rate (TPR) of 0.33 (9 out of 27 experimental interaction loss events are correctly predicted), and a false positive rate (FPR) of 0.2 (3 out of 15 experimental interaction retention events are incorrectly predicted). A random predictor would give a TPR equal to the FPR whereas a TPR that is larger than the FPR indicates that predictions are better than random. Our observed TPR is larger than the observed FPR, however the difference is not statistically significant due to small sample size (p = 0.48, two-sided Fisher’s exact test). To increase the sample size, we expanded the 34 reference interactions with full DDI annotations in the dataset of Yang et al. (2016) by including 226 additional reference interactions with partial DDI annotations for a total of 260 domain-annotated reference interactions. A reference interaction has a partial DDI annotation if a reference protein with multiple isoforms contains an interacting domain of a DDI whereas its interaction partner does not contain the other interacting domain of the DDI. An alternative isoform of the reference protein is then predicted to lose the interaction if it loses all of its interacting domains, otherwise the interaction is retained. Using this method, we predicted isoform-specific interactions from these 260 domain-annotated reference interactions, and compared our predictions with experiments. In terms of predicting interaction loss events, we obtained a true positive rate (TPR) of 0.31 (53 out of 171 experimental interaction loss events are correctly predicted) and a false positive rate (FPR) of 0.10 (12 out of 117 experimental interaction retention events are incorrectly predicted). Similar to the rates obtained above, the observed TPR here is three times larger than the observed FPR and the difference is statistically significant due to much larger sample size (p = 2.6 x 10^−5^, two-sided Fisher’s exact test), indicating that our prediction method is of high-quality, performing significantly better than random predictions. Since the benchmark experimental dataset may contain errors, the actual TPR of our method is expected to be even higher than the observed TPR.

## Discussion

Our computational method for predicting isoform-specific interactions is reliable because it does not aim to predict new PPIs from scratch. Rather, it starts with the annotation of experimentally determined PPIs with known DDIs, and predicts the retention or loss of PPIs based on the retention or loss of the annotated DDIs in different isoforms. All these individual steps are expected to generate high-quality predictions; thus our overall isoform-interaction predictions are expected to be of high quality as well. Indeed, the high quality of our predictions is confirmed by experimental validations. At the same time, our method is limited in that it can only predict isoform interactions from known reference PPIs. If a reference protein does not have any interactions in the reference interactome, it is not possible to predict interactions for any of its alternative isoforms. Therefore, the size of the isoform interactome predicted by our method is ultimately constrained by the size of the reference interactome.

Since our method predicts isoform interactions based on experimentally determined interactions between reference proteins, it is important to ensure the high quality of these experimentally determined interactions. Interactions in the HI-II-14 reference binary interactome are of high-quality since they were subjected to multiple screening and several other quality-control measures. On the other hand, interactions in the IntAct reference interactome were curated from different sources with varying quality. Furthermore, IntAct may contain indirect interactions between proteins in the same complex. To ensure the high quality of the IntAct-derived interactions, we only included physical interactions in IntAct reported by at least two independent experimental studies. In addition, our DDI-annotated interactome significantly enriches for direct binary interactions and filters out indirect interactions, as indirect interactions are much less likely to be annotated with DDIs than direct binary interactions.

For reference PPIs with multiple DDI annotations, our method imposes a strict requirement that for an interaction to be lost, all DDI annotations of that interaction must be lost, otherwise the interaction is retained. Assuming that the interactivity of different domains is independent of each other within the same protein, this strict requirement maximizes the accuracy of predicted lost interactions, at the cost of possibly reducing the accuracy of predicted retained interactions. It should be noted that this is not a significant problem in our study, as about half of the PPIs in the HI-II-14 and IntAct domain-resolved interactomes have only one DDI annotation (51% and 46%, respectively).

While our predicted isoform interactomes reveal extensive network remodeling by AS, the ratio of remodeled protein pairs (i.e., the ratio of the number of protein pairs interacting with different subsets of isoforms of the same gene to the number of protein pairs interacting with the same subset of isoforms of the same gene) is different in the two predicted isoform interactomes (12.9% in IntAct, and 4.4% in HI-II-14). This difference is due to systematic differences in the types of interactions reported in the two reference interactomes and in the fraction of protein hubs between the two domain-resolved interactomes. Since proteins with significant medical and scientific interests are intensely studied in the literature, protein hubs are much better annotated with domains in the literature-curated IntAct interactome than in the systematic HI-II-14 interactome. Indeed, protein hubs with vertex degree >23 (~5% of proteins in both binary interactomes) are 3.4 times more likely to have a domain-annotated interaction in the IntAct interactome than in the HI-II-14 interactome, whereas average proteins are only 1.6 times more likely to have a domain-annotated interaction in the IntAct interactome than in the HI-II-14 interactome. As a result, the fraction of protein hubs is significantly larger in the IntAct domain-resolved interactome than in the HI-II-14 domain-resolved interactome (11.1% in IntAct and 5% in HI-II-14, for hubs with vertex degree >5). A larger fraction of protein hubs in the IntAct domain-resolved interactome is the major cause for the observed larger ratio of remodeled protein pairs in the IntAct isoform interactome, since protein pairs interacting with different subsets of isoforms of the same gene can only be created by isoforms that lose some but not all interactions (which is more likely to occur for isoforms of a protein hub with many interactions), and cannot be created by isoforms that lose all interactions at once (which is more likely to occur for isoforms of a protein non-hub with few interactions). Indeed, the observed difference (12.9% in IntAct, and 4.4% in HI-II-14) in the ratio of remodeled protein pairs between the two isoform interactomes is almost completely eliminated (6.1% in IntAct, and 5.2% in HI-II-14) upon the removal of all protein hubs with vertex degree >5. This observed difference does not significantly bias our isoform-specific interaction predictions for the following reasons: we predict isoform-specific interaction retention/loss one isoform at a time based on sequence information only; no significant differences are observed between the two isoform interactomes (IntAct and HI-II-14) in terms of the fraction of isoforms per gene losing at least one interaction (8.2% and 8.6%) and the fraction of isoform pairs per gene with different interaction profiles (14.2% and 15.3%); we only draw conclusions from comparing protein pairs in terms of their biological properties, and from observations that are consistent between the two isoform interactomes.

Detailed sequence information is available for some reference proteins, but not others. When the precise sequence information is not available, we chose the reference isoform designated by UniProt to represent each reference protein in the reference interactomes. These UniProt-designated reference isoforms are typically either the longest isoform (~88%) or the most prevalent isoform; hence they are most likely the ones used in different experimental studies to map the reference interactions. In the unlikely event that some reference proteins in the reference interactomes are represented by a different isoform than the one designated by UniProt, our method only requires the knowledge that a reference PPI exists, and can still work well without knowing the exact sequence of the reference protein. Given the experimental knowledge that a reference PPI exists, our method predicts that an alternative isoform loses this interaction if and only if the isoform loses all possible interacting domains present in the UniProt-designated reference isoform with typically the longest sequence, which in half of the cases is just one interacting domain. Hence, our predictions of interaction loss and retention remain very reasonable even in the presence of minor discordances in reference isoform annotations.

In summary, we developed a domain-based computational method for predicting an isoform interactome from a reference interactome by integrating structural domain information with experimentally determined interactions. Our predictions reveal extensive remodeling of the human interactome network by AS: ~22% of genes with two or more isoforms in the predicted isoform interactome have at least one isoform losing an interaction, and ~18% of isoform pairs encoded by the same gene in the isoform interactome network have different interaction profiles. Our isoform-interaction prediction framework is of high quality as it performs significantly better than random predictions when assessed by experimental data. In addition, our predicted isoform interactome is larger and probes a different part of the isoform space than the experimental isoform interactome of Yang et al. (2016) [34]. In terms of the space of genes with at least two isoforms tested for interactions, our predicted isoform interactomes cover ~4 times larger gene space than Yang et al. (2016). Only ~19% of the gene space covered by our predicted HI-II-14 isoform interactome is covered by Yang et al. (2016), and only ~8% of the gene space covered by our predicted IntAct isoform interactome is covered by Yang et al. (2016). Despite this minimal overlap, the biological insights provided by our predicted isoform interactome are largely consistent with Yang et al. (2016). Compared to protein pairs interacting with the same subset of isoforms of the same gene, protein pairs interacting with different subsets of isoforms of the same gene tend to be more divergent in biological function, disease phenotype, and tissue expression. Thus, our computational study complements large-scale experimental efforts on mapping the human isoform interactome, and highlights the broad applicability of AS-mediated interactome remodeling as a driving force for the functional divergence of different isoforms encoded by the same gene.

## Methods

### Domain-resolved annotation of the reference interactome

Domain-domain interactions (DDIs) were retrieved from the 3did Database of Three-Dimensional Interacting Domains [35] (retrieved May 2017) and the DOMINE Database of Protein Domain Interactions [36] (retrieved Oct 2015). For DOMINE DDIs, we kept the 6,634 DDIs inferred from Protein Data Bank (PDB) entries, and excluded those DDIs predicted by computational methods. We then combined the 6,634 PDB-inferred DDIs from DOMINE with the 10,593 PDB-inferred DDIs from 3did. After removing duplicates, we obtained a total of 11,557 DDIs. To annotate PPIs with DDIs, we first annotated proteins in the reference interactome with structural domains. Gene Entrez IDs in the HI-II-14 reference interactome were mapped to Swiss-Prot IDs using the Retrieve/ID mapping tool provided by UniProt [37]. After removing self-interactions, 4,091 genes with unique SwissProt IDs were then used for further analysis. Swiss-Prot IDs for genes in the IntAct reference interactome were provided by the IntAct database. We then retrieved protein sequences from UniProt and scanned them for Pfam domains using HMMER hmmscan [57] with an E-value cutoff of 10^−5^. After annotating each protein in the reference interactome with its structural domains, each PPI in the reference interactome was annotated with a DDI if one of the interacting proteins was annotated with an interacting domain of the DDI and the other interacting protein was annotated with the other interacting domain of the same DDI. Only PPIs annotated with at least one DDI were included in the domain-resolved interactome.

### Predicting isoform interactions from the domain-resolved reference interactome

Alternative isoforms of each reference protein in the domain-resolved reference interactome were annotated with structural domains by retrieving their sequences from UniProt and scanning them for Pfam domains using HMMER hmmscan [57] with an E-value cutoff of 10^−5^. Then, for each interaction between two reference proteins in the domain-resolved reference interactome annotated with one or more DDIs, we predicted that an alternative isoform of one protein loses its interaction with the other protein if the isoform interaction loses all the above-mentioned DDI annotations. If the isoform interaction keeps at least one of the DDI annotations, the interaction was predicted to be retained.

### Calculating Gene Ontology similarity for pairs of proteins

We retrieved Gene Ontology (GO) associations from the UniProt-GOA database [38] (retrieved Feb 2016), which provides a set of 16,329 controlled hierarchical GO terms split into three categories: 3,812 molecular function terms, 11,042 biological process terms, and 1,475 cellular component terms. GO terms were mapped onto reference proteins using the Swiss-Prot IDs associated with each GO term. We quantified GO similarity between two proteins by calculating the Jaccard similarity index of their GO association profiles, which is defined as the number of GO terms shared by both proteins divided by the number of GO terms associated with at least one protein. Similarly, we calculated molecular function similarity, biological process similarity and cellular component similarity between two proteins by calculating the Jaccard similarity index of their GO association profiles using the corresponding GO entries.

### Calculating disease similarity for pairs of proteins

We retrieved gene-disease associations from the DisGeNET database [39, 40] (retrieved July 2016), which integrated data from UniProt [37], ClinVar [58], Orphanet (http://www.orpha.net), CTD [59], and the GWAS Catalog [60]. Diseases were mapped onto reference proteins by mapping disease-associated gene names to Swiss-Prot IDs using the mapping table provided by DisGeNET. To calculate the fraction of disease subnetworks shared by two proteins, we included in each protein’s disease association profile all diseases associated with that protein and its first-degree neighbors in the HI-II-14 reference binary interactome. We then used the Jaccard similarity index to calculate the fraction of disease subnetworks shared by the two proteins, where two proteins share a specific disease subnetwork if each of the two proteins or any of its interaction partners in the HI-II-14 reference binary interactome is annotated with that disease.

### Calculating tissue co-expression for pairs of proteins

We used the RNA-Seq dataset of Illumina Body Map 2.0 [43] (retrieved Jan 2016), normalized using log2 transformation, to quantify gene expression levels in 16 human body tissues: adipose, adrenal, brain, breast, colon, heart, kidney, leukocyte, liver, lung, lymph node, ovary, prostate, skeletal muscle, testis and thyroid. Gene expression profiles were mapped onto reference proteins in the IntAct reference interactome by mapping the protein Swiss-Prot IDs to gene names using the Retrieve/ID mapping tool provided by UniProt [37]. Gene expression profiles were mapped onto reference proteins in the HI-II-14 reference interactome using gene names provided by the original HI-II-14 dataset. Tissue co-expression of each pair of reference proteins was calculated as Pearson’s correlation coefficient of their gene expression profiles.

### Domain-resolved annotation of the experimental isoform interactome dataset

The quality of our computational method was assessed by the experimental isoform interactome dataset of Yang et al. (2016) [34], which consists of 985 interactions and 763 non-interactions between reference proteins taken from the human ORFeome V8.1 database [61] and newly-cloned isoform sequences. 310 of these interactions involve an ORFeome protein and a newly-cloned reference isoform sequence (reference-reference), and the rest of the interactions involve an ORFeome protein and a newly-cloned alternative isoform sequence (reference-alternative). We annotated all isoforms in this experimental dataset with structural domains by scanning their sequences for Pfam domains using HMMER hmmscan [57] with an E-value cutoff of 10^−3^. The 11,557 PDB-inferred DDIs from 3did [35] and DOMINE [36] were then used to annotate the reference-reference interactions. A protein-protein interaction was given a full DDI annotation if one protein was annotated with an interacting domain of a DDI, and its interaction partner was annotated with the other interacting domain of the same DDI. A protein-protein interaction was given a partial DDI annotation if one protein with multiple isoforms was annotated with an interacting domain of a DDI, even if the interaction partner was not annotated with the other interacting domain of the same DDI.

### Web tool for querying our predicted human isoform interactome

We have created a web tool called “DIIP: Domain-based Isoform Interactome Prediction” that allows users to query our predicted isoform interactome for isoform-specific interactions of a protein of interest. In addition, our web tool gives users a second advanced option to predict isoform-specific interactions using our isoform interactome prediction method (DIIP) from interactions provided by the user. Interactions provided by the user do not need to be part of our predicted isoform interactome. The web tool can be accessed at the following URL: http://bioinfo.lab.mcgill.ca/resources/diip. The code used for predictions and analysis is available at the following URL: http://github.com/MohamedGhadie/isoform_interactome_prediction.

## Supporting information

S1 Table HI-II-14 domain-resolved reference interactome. All interactions in the HI-II-14 domain-resolved reference interactome with their DDI annotations.
(XLSX)

S2 Table IntAct domain-resolved reference interactome. All interactions in the IntAct domain-resolved reference interactome with their DDI annotations.
(XLSX)

S3 Table Predicted HI-II-14 isoform interactome. All known and predicted interactions in the predicted HI-II-14 isoform interactome. Known interactions are the experimentally observed interactions also belonging to the HI-II-14 domain-resolved interactome. Predicted interactions are isoform interactions predicted to be either retained or lost.
(XLSX)

S4 Table Predicted IntAct isoform interactome. All known and predicted interactions in the predicted IntAct isoform interactome. Known interactions are the experimentally observed interactions also belonging to the IntAct domain-resolved interactome. Predicted interactions are isoform interactions predicted to be either retained or lost.
(XLSX)
